# Patterns of Healthcare Use and Disease Burden Among Older Adults in Poland: A Large-Scale Retrospective Study of Primary Care Utilization

**DOI:** 10.3390/geriatrics10060150

**Published:** 2025-11-13

**Authors:** Krzysztof Marcin Zakrzewski, Paulina Mularczyk-Tomczewska, Tytus Koweszko, Łukasz Czyżewski, Andrzej Silczuk

**Affiliations:** 1 The Independent Group of Public Ambulatory Care Institutions Warsaw-Ochota, 02-962 Warsaw, Poland; 2 Department of Public Health, Faculty of Life Sciences, Medical University of Warsaw, 02-091 Warsaw, Poland; paulina.mularczyk-tomczewska@wum.edu.pl; 3 Department of Community Psychiatru, Faculty of Life Sciences, Medical University of Warsaw, 02-091 Warsaw, Poland; tytus.koweszko@wum.edu.pl (T.K.); andrzej.silczuk@wum.edu.pl (A.S.); 4 Department of Geriatric Nursing, Faculty of Life Sciences, Medical University of Warsaw, 02-091 Warsaw, Poland; lukasz.czyzewski@wum.edu.pl

**Keywords:** aging population, older adults, healthcare utilization, chronic disease, multimorbidity, polypharmacy, COVID-19 pandemic, telemedicine, mental health services

## Abstract

**Background**: Population aging in Poland has led to rising healthcare needs, but comprehensive evidence on long-term utilization patterns is limited. This study aimed to analyze healthcare use and disease burden among older adults, with particular focus on the impact of the COVID-19 pandemic, including its effects on care pathways, continuity of services, and differences between urban and rural populations. **Methods**: We conducted a retrospective study using anonymized medical records from a primary healthcare network in Poland between January 2020 and December 2024. The sample included 42,844 patients aged 60 years or older patients aged ≥60 years, with a total of 738,300 consultations. Data on demographics, visit type, prescriptions, referrals, diagnostic tests, and follow up were analyzed using chi-square tests, logistic regression, and nonparametric comparisons. **Results**: The mean age of patients was 77.5 years (SD 9.4), and 63.7% were women. The mean number of visits in the preceding 12 months was 10.3 (range 0–460). Prescriptions were issued in 56.9% of visits, referrals in 33.5%, and diagnostic tests in 21.4%. Patients with chronic diseases were more likely to receive diagnostic tests than those without (32.4% vs. 21.1%, χ^2^ = 1570.42, *p* < 0.0001), but less likely to return within 30 days (23.4% vs. 39.4%, χ^2^ = 2243.76, *p* < 0.0001). First visits generated more referrals than follow ups (41.6% vs. 32.9%, χ^2^ = 1620.51, *p* < 0.0001). Completed visits were strongly associated with prescription issuance (63.2% vs. 16.4%, χ^2^ = 1048.76, *p* < 0.0001). Return analyses showed that patients with more prior visits were significantly more likely to re-engage at 30, 60, and 90 days (all *p* < 0.0001). Age correlated positively with total number of visits (ρ = 0.136, *p* < 0.05) with the oldest adults more likely to return at 60 and 90 days. Visit realization decreased during early pandemic phases but increased significantly post-COVID (OR = 1.76, *p* < 0.001). Psychiatric referrals increased the odds of return within 30 days (OR = 1.42) and 60 days (OR = 1.36). **Conclusions**: Older adults in Poland demonstrate high healthcare utilization, with patterns shaped by age, chronic disease status, and pandemic-related disruptions. While statistical associations were robust, effect sizes were small, highlighting the need to focus on clinically meaningful differences in planning geriatric care. The findings highlight that COVID-19 acted as a major modifying factor of healthcare engagement, revealing the vulnerability of geriatric care continuity to system-level disruptions.

## 1. Introduction

Demographic change in Europe, including Poland, is characterized by declining population numbers and a rising share of older adults [1,2]. Classic and health adjusted dependency metrics show that age based measures can overstate the burden, but they consistently indicate growing needs in health and social care [1]. Regional analyses demonstrate that different European regions show different directions and rates of change over time, rather than following a single unified pattern [2]. Aging is associated with greater demand for injury prevention and rehabilitation among older people [3], while sustained life expectancy gains in Poland mirror broader European trends [4]. Migration interacts with aging and shapes health needs and integration policies [5].

In Poland, increases in life expectancy and the growing number of older adults have not been matched by proportional improvements in healthy aging. Clinical and functional risks are well documented, including falls and reduced independence [6], high prevalence of chronic illness and disability [7], notable rates of polypharmacy across Europe [8], and social and biomedical determinants of activity limitations in Polish cohorts [9]. At the macro level, modeling links population aging, migration, and productivity [10]. Reproductive health literacy and fertility awareness provide context for sustained low fertility [11]. Adverse outcomes of polypharmacy are established [12], national data quantify its prevalence and age structure [13], and practical management approaches are available [14].

Mental health burdens, including loneliness, are common in older adults [15]. Large national cohorts in Poland show high polypharmacy in older age [16]. System pressures intensified during the pandemic and are reflected in access to care and dependency indicators [17], and alternative measures such as the health adjusted dependency ratio provide a complementary lens [18]. Multimorbidity is closely linked to disability [19], and mortality profiles in older age have continued to shift [20]. Fertility postponement and sub replacement fertility remain important drivers of demographic aging in Europe and Poland [21,22].

The COVID-19 pandemic transformed both healthcare delivery and patient behavior, especially among adults aged 60 and older. Telehealth adoption rose sharply in this group, increasing from about 5 percent before the pandemic to about 21 percent during the pandemic. Its uptake was strongly conditioned by access to devices, internet connectivity, and technology learning [23]. In Poland, the use of publicly financed services fell markedly in 2020, with only partial rebound in 2021, highlighting structural constraints in service organization and financing [24].

Across Europe, comparative SHARE analyses show that remote consultations among adults aged 50 and older varied by health system characteristics and individual resources. They were least common in the oldest group [25]. Clinicians reported that telehealth improved timeliness and care coordination for older patients. However, they also highlighted barriers linked to rural residence, cognitive impairment, lower literacy, and concerns about sustainability and reimbursement [26].

Reviews caution that telemedicine may unintentionally amplify disparities unless digital access and usability are actively addressed [27]. In Poland, patient surveys confirmed rapid uptake of telephone-based consultations, but also pointed to mixed satisfaction and persistent usability barriers during the first two pandemic years [28]. Determinant studies in older adults further indicate that psychological need and health limitations are major drivers of telemedicine use, which has implications for post-pandemic planning and targeted support [29].

Despite the urgency of these issues, scientific literature still provides limited large-scale data on older populations. Existing studies often focus on small cohorts, narrow time frames, or specific sectors of healthcare, which restricts their representativeness and applicability to policy planning. In particular, little is known about long-term patterns of healthcare utilization across multiple services, such as primary care, specialist consultations, psychiatry, rehabilitation, and dental care.

The present study addresses this gap by conducting a large-scale retrospective analysis of electronic health records from a territorially dispersed medical institution covering both urban and rural populations. The objective of this study was to analyze primary care utilization and disease burden among older adults in Poland, with a particular focus on how the COVID-19 pandemic altered care pathways, access to services, and continuity of follow-up.

## 2. Materials and Methods

### 2.1. Study Design and Setting

This study used a retrospective observational design based on anonymized electronic health records (EHRs) from a territorially dispersed primary healthcare network in Poland. The observation period spanned from January 2020 to December 2024 and was intentionally selected to cover the full course of the COVID-19 pandemic and the initial post-pandemic phase. This allowed comparison of healthcare utilization before, during, and after pandemic-related system disruptions.

### 2.2. Data Source

Data were extracted from the network’s EHR system using predefined structured fields. Only routinely collected administrative and clinical records were used. No manual chart review or additional coding was performed. The dataset included primary care, specialist outpatient services, psychiatry and addiction treatment, medical rehabilitation, and dental care.

### 2.3. Study Population

The study population comprised all patients aged ≥60 years who had at least one registered consultation within the study period. No additional exclusion criteria were applied, ensuring complete coverage of older adults receiving care in the network. In total, 738,300 consultations from 42,844 unique patients were analyzed. Patients were categorized into early senior age (60–74 years, “young old”), advanced senior age (75–89 years, “old old”), and long-lived individuals (≥90 years, “oldest old”) in line with international gerontological classification.

### 2.4. Variables and Outcomes

For each consultation, demographic (age, sex), temporal (date of visit, pandemic period), and service-related data were extracted. Primary outcomes were prescription issuance, specialist referral, diagnostic test ordering, and visit completion. Follow-up behavior was assessed at 30, 60, and 90 days as a secondary outcome reflecting continuity of care.

### 2.5. Data Quality and Missingness

Missing and incomplete data were assessed prior to analysis. Because missingness was minimal and limited to non-clinical administrative variables (e.g., visit status coding), no imputation procedures were applied. Listwise exclusion was used for analyses requiring complete information for a given model.

### 2.6. Statistical Analysis

Descriptive statistics were used to characterize the study population and consultation patterns. Chi-square tests with Yates’ correction were applied to examine associations between categorical variables. Logistic regression models were used to explore predictors of service use and follow-up. Temporal comparisons across pre-pandemic, pandemic, and post-pandemic phases were assessed using the McNemar test. Statistical analyses were performed in Statistica 13.3 (StatSoft Inc., Tulsa, OK, USA), with significance set at *p* ≤ 0.05.

## 3. Results

The analytic dataset comprised 738,300 visits of 42,844 patients aged 60 years or older, who together accounted for over one million consultations across primary, specialist, psychiatric, rehabilitation, and dental services. Table 1 presents the main characteristics of the study cohort, including demographic distribution, burden of chronic conditions, and key indicators of healthcare use such as prescription issuance, referrals, diagnostic tests, and follow-up visits.

### 3.1. Association Between Chronic Disease and Diagnostic Test Ordering

A chi-square test of independence was conducted to assess the relationship between chronic disease diagnosis and the likelihood of receiving diagnostic test orders. The results were statistically significant (χ^2^(1) = 1570.42; *p* < 0.0001), indicating that patients with chronic diseases were more likely to have diagnostic tests ordered (32.4%) compared to those without chronic conditions (21.1%). The phi coefficient (φ = 0.00213) suggests a very weak effect size, likely due to the substantial imbalance in group sizes. While the results may have practical implications for the prevention and monitoring of chronic patients, they should be interpreted with caution.

### 3.2. Association Between Visit Type and Referral Issuance

A chi-square test of independence was conducted to examine the relationship between visit type (first vs. follow-up) and referral issuance. The results were statistically significant (χ^2^(1) = 1620.51; *p* < 0.0001), indicating that referrals were more frequently issued during first visits (41.6%) compared to follow-up visits (32.9%). The phi coefficient (φ = 0.00219) suggests a very weak effect size, likely due to the substantial imbalance in group sizes. This finding is counterintuitive, as first visits are expected to result in fewer referrals; however, it may reflect the need for diagnostics during initial encounters. Despite the statistical significance, the effect size is very weak, warranting careful consideration of its clinical and practical implications.

### 3.3. Association Between Visit Completion Status and Prescription Issuance

A chi-square test of independence was conducted to assess the relationship between visit completion status and prescription issuance. The results were statistically significant (χ^2^ = 1048.76; df = 1; *p* < 0.0001), indicating a strong association between visit type and prescription frequency. Prescriptions were issued more frequently during completed visits (63.2%) compared to declared-only visits (16.4%). The phi coefficient (φ = 0.00194) suggests a very weak effect size, likely due to the substantial imbalance in group sizes.

### 3.4. Return Analysis: Impact of Prior Visits on Short-Term Return Rates

Across all intervals (30, 60, and 90 days), patients who returned had significantly more visits in the past 12 months (all *p* < 0.0001), as demonstrated by Mann–Whitney U tests. The strength of association increased over time (Z = −307.13 to −393.52), indicating a consistent relationship between recent healthcare engagement and the likelihood of short-term return. These findings underscore the importance of prior visit history as a strong predictor of follow-up behavior.

### 3.5. Association Between Chronic Disease and 30-Day Return Visits

A chi-square test of independence was conducted to examine the relationship between chronic disease diagnosis and 30-day return visits. The results were statistically significant (χ^2^(1) = 2243.76; *p* < 0.0001), indicating a relationship between chronic condition status and short-term follow-up. Patients without chronic conditions returned within 30 days more frequently (39.4%) than those with chronic conditions (23.4%). The phi coefficient (φ = 0.00304) suggests a very weak effect size, likely due to the substantial imbalance in group sizes. This finding is unexpected, as patients with chronic diseases were anticipated to return more often; this may reflect a more distributed care schedule or stabilization of treatment. Despite the statistical significance, the effect size is very weak, warranting consideration of its practical and clinical implications.

### 3.6. Impact of Age on Return Probability

Age at visit was significantly associated with follow-up timing across all intervals. Patients who returned within 30 days were statistically younger than those who did not (U = 61,288,668,535; Z = −39.40; *p* < 0.0001), while those returning within 60 days (U = 62,385,231,744; Z = −56.68; *p* < 0.0001) and 90 days (U = 56,938,517,518; Z = −59.79; *p* < 0.0001) were significantly older than their respective non-returning counterparts. These findings suggest a temporal shift in return patterns: younger patients tend to return sooner, whereas older individuals are more likely to engage in mid- and long-term follow-up, potentially reflecting structured care pathways or chronic condition management.

### 3.7. Association Between Patient Sex and Healthcare Issuance

Statistical analyses revealed significant associations between patient sex and the issuance of prescriptions (χ^2^(1) = 12.74, *p* = 0.0004), referrals (χ^2^(1) = 91.66, *p* < 0.0001), and diagnostic test orders (χ^2^(1) = 7.91, *p* = 0.0049). In each case, women received slightly more interventions than men (e.g., prescriptions: 57.1% vs. 56.7%), but effect sizes were negligible (φ < 0.0002), indicating minimal practical relevance. These findings suggest that while sex-based differences are statistically detectable, they are unlikely to reflect meaningful clinical disparities.

### 3.8. Association Between Place of Medical Appointment and Healthcare Issuance

Statistical analyses revealed significant associations between the place of medical appointment (urban vs. suburban) and the issuance of prescriptions (χ^2^ = 769.96, *p* < 0.0001), referrals (χ^2^ = 1004.33, *p* < 0.0001), and diagnostic test orders (χ^2^ = 397.44, *p* < 0.0001). In each case, patients from suburban areas received slightly more interventions than those from urban areas (e.g., prescriptions: 61.8% vs. 56.4%), yet effect sizes were consistently weak (φ < 0.0014). These findings, while statistically robust, suggest limited clinical relevance and may reflect structural factors such as reduced access to specialists or differing care pathways in suburban settings.

### 3.9. Association Between Patient Sex and Chronic Disease Diagnosis

A chi-square test of independence was conducted to examine the relationship between patient sex and chronic disease diagnosis. The result was not statistically significant (χ^2^(1) = 3.13; *p* = 0.0768), indicating no significant relationship between sex and chronic condition status. Although the proportion of chronic diagnoses was slightly higher among women (2.88%) than men (2.95%), this difference did not reach statistical significance. Consequently, the findings suggest that there is no meaningful association between sex and the likelihood of receiving a chronic disease diagnosis.

### 3.10. Impact of Time Period on Visit Realization

Logistic regression revealed a significant association between time period and visit realization (χ^2^ = 145.14, *p* < 0.0001), with each successive phase increasing the odds of realization by 13% (OR = 1.13). This trend likely reflects systemic recovery and institutional adaptation following early pandemic disruptions. A combined model confirmed that both time period and age independently predicted realization (χ^2^ = 145.95, *p* < 0.0001), with slightly higher odds among older patients. While effects were statistically robust, their practical relevance may depend on contextual factors such as service type and patient profile.

### 3.11. Impact of Psychiatric/Psychological Referral on Return Visits

Psychiatric or psychological referrals to mental health specialists were significantly associated with increased odds of return within 30 days (OR = 1.42, *p* < 0.001) and 60 days (OR = 1.36, *p* < 0.001), as assessed in logistic regression models using 738,300 valid cases. However, the effect diminished over time and was not statistically significant at 90 days (OR = 1.16, *p* = 0.075). These findings suggest that referrals to mental health specialists may effectively encourage short- and mid-term follow-up visits, but their influence on long-term engagement appears limited.

### 3.12. Association Between Psychiatric/Psychological Referral and Predictors

A multivariate logistic regression revealed significant associations between psychiatric/psychological referral and sex, age, and pandemic period (χ^2^(4) = 491.44, *p* < 0.001; N = 738,300). Men were nearly twice as likely to be referred as women (OR = 1.90), while referral likelihood decreased with age (OR = 0.93) and was lower during the pandemic period (OR = 0.89). Location was not a significant predictor (*p* = 0.943), with unstable estimates suggesting sparse data. These findings underscore the role of demographic and temporal factors in shaping referral patterns, though caution is advised in interpreting the geographic effects.

### 3.13. Impact of Age, Location, Sex, and Pandemic Period on Visit Realization

Multivariate logistic regressions across pandemic phases revealed consistent effects of location and sex on visit realization. Patients from suburban areas and male patients were more likely to realize visits (OR ≈ 1.47 and OR ≈ 1.14, *p* < 0.001), while age showed a modest negative association in earlier phases (OR ≈ 0.996), becoming non-significant post-Third Wave. Visit realization was markedly reduced during threat states and early waves (ORs ranging from 0.36 to 0.78) but significantly increased in the post-COVID period (OR = 1.76, *p* < 0.001), suggesting systemic recovery. Geographic and gender effects remained stable, while age and temporal dynamics varied across phases.

### 3.14. Relationship Between Age and Number of Visits in the Past 12 Months

A Spearman rank-order correlation was conducted to examine the relationship between patient age at visit and the number of visits in the past 12 months. The correlation coefficient was ρ = 0.136 (*p* < 0.05), indicating a statistically significant but weak positive association. This suggests that older patients tend to have more frequent healthcare visits, although the strength of the relationship is modest (Table 2).

## 4. Discussion

This large-scale retrospective study offers novel insights into healthcare utilization among older adults in Poland across the pre-pandemic, pandemic, and post-pandemic periods. By analyzing more than one million consultations, we were able to identify consistent patterns of service use, demographic and clinical predictors of engagement, and the systemic effects of the COVID-19 crisis on access and continuity of care. The findings highlight the complexity of healthcare demand in aging societies and underscore the importance of tailoring strategies to different age groups, disease profiles, and service settings.

The overall number of visits, averaging more than ten per patient per year, reflects the considerable healthcare burden of older adults in Poland. This observation aligns with earlier national studies documenting high prevalence of chronic diseases, disability, and activity limitations among older cohorts [7,9]. Moreover, the widespread use of multiple medications, described in European and Polish studies [8,13,16], provides further evidence that polypharmacy and multimorbidity are key drivers of healthcare demand. In our dataset, patients with chronic conditions were more likely to receive prescriptions and diagnostic tests, confirming the clinical need for intensified monitoring and long-term management. Such findings are consistent with prior evidence that polypharmacy is not only frequent but also associated with adverse outcomes in older populations [12].

Unexpectedly, patients without chronic conditions were more likely to return for follow-up within thirty days, whereas those with chronic diseases tended to return later. This may reflect differences in care organization: individuals with established chronic conditions are often scheduled for longer-term monitoring, while patients without such diagnoses may seek acute or subacute follow-up. Prior studies demonstrated that multimorbidity in elderly patients is strongly associated with disability and long-term healthcare needs rather than frequent short-term encounters [19]. Our data also revealed an age-related gradient: Younger seniors were more likely to return quickly, whereas the oldest groups were more engaged in medium and long-term follow-up. This suggests that structured chronic care pathways may dominate service use in the oldest cohorts. National mortality and disability data confirm that healthcare needs escalate with age, but the structure of care shifts accordingly [7,20].

The observed pattern, in which patients without chronic conditions returned more frequently within 30 days, may be related to the organization of care pathways. Individuals with established chronic diagnoses usually receive structured long-term follow-up, whereas those without a confirmed chronic condition may perceive a greater need for short-term reassurance or diagnostic clarification. Differences in perceived urgency, trust in initial management, or fragmentation of care may contribute to the higher rate of early returns among patients without chronic disease, and existing evidence suggests that such trust-related mechanisms are partly shaped by broader attitudes toward care and mental health in older populations [30,31].

Sex and geographic differences were present but modest. Women received slightly more prescriptions, referrals, and diagnostic tests than men, which is in line with broader European observations of greater healthcare engagement among women [8]. Patients from suburban areas received more interventions than those from urban areas, possibly reflecting limited access to specialist services and compensatory prescribing by primary care physicians. Similar geographic disparities have been reported in Polish healthcare utilization analyses, especially during pandemic-related disruptions [24]. However, the effect sizes in our study were small, suggesting that these patterns are of limited clinical relevance but may still indicate structural inequities in service delivery.

The COVID-19 pandemic had a profound effect on service realization. We observed marked reductions during the early waves and threat states, followed by gradual recovery in the post-COVID period. These results echo findings from European surveys documenting widespread disruptions in access for older adults [17] and from Polish national analyses showing declines in 2020 with only partial rebound in subsequent years [24]. More recent reviews have emphasized that continuity of care for patients with chronic diseases was frequently interrupted due to system-level reorganizations, logistical barriers, and altered clinical pathways [32]. Such factors may partly explain why our study found less frequent short-term returns among patients with chronic conditions. These shifts indicate not only temporary access barriers but also a reconfiguration of care pathways, with delayed or deferred care becoming embedded in utilization patterns even after restrictions eased.

Mental health referrals played a notable role in continuity of care. Patients referred to psychiatric or psychological services were more likely to return within 30 and 60 days, though the effect diminished at 90 days. This suggests that integrating mental healthcare into geriatric practice may improve short-term engagement but sustaining long-term follow-up remains challenging. International reviews of pandemic responses showed that countries which rapidly adapted their mental health services delivery, for example, through hybrid models or extended hours, maintained better continuity [33]. Furthermore, a multicountry interrupted time series demonstrated substantial fluctuations in the number of primary care mental health visits during the pandemic, underscoring the vulnerability of this sector to systemic shocks [34]. Our findings support the view that embedding mental health services into primary and geriatric care could strengthen continuity and resilience.

Telemedicine adoption is another important dimension. Although prior surveys in Poland reported rapid uptake of remote consultations [28], patient experiences were mixed, and usability barriers persisted. Comparative analyses across Europe revealed that telehealth use among older adults was uneven, with younger seniors and individuals with stronger digital resources more likely to engage [23,25]. Recent evidence from Germany confirmed that advanced age, lower education, and rural residence reduce the likelihood of telemedicine use [35]. Qualitative studies further suggest that both older patients and physicians often perceive teleconsultations as inferior to in-person visits due to the lack of visual cues and reduced diagnostic trust [36]. These findings contextualize our observations of fluctuating visit realization across pandemic phases, indicating that digital inequalities and perceptions of reduced quality may have limited the compensatory potential of telemedicine. It is also important to strengthen public awareness both in the field of mental health literacy and in self-monitoring among older adults, where a marked gap has been observed between younger generations and the senior population [37,38]. This underscores that telemedicine alone cannot compensate for structural gaps in geriatric care unless accompanied by targeted digital support and user-specific adaptations.

Several strengths of this study should be emphasized. The dataset was large, territorially diverse, and covered multiple phases of the pandemic, allowing for robust analyses across service types and demographic subgroups. The inclusion of both urban and suburban populations enhances representativeness. However, limitations must also be acknowledged. The reliance on a single healthcare network may restrict generalizability. Chronic conditions were recorded in a binary manner, underestimating the complexity of multimorbidity. Very large sample sizes increased the likelihood of detecting statistically significant but clinically negligible associations, requiring careful interpretation of effect sizes. Finally, the observational design precludes causal inference. In addition, routine electronic medical records may be affected by information bias or occasional coding inaccuracies, which could influence variable classification. Finally, due to the observational and retrospective design, the study cannot establish causal relationships. Taken together, these findings show that the pandemic did not merely disrupt service volume but also altered the behavioral dynamics of healthcare engagement in this age group.

Despite these limitations, the study may provide valuable evidence for healthcare planning in aging societies. The results underscore the need to tailor follow-up strategies to different age groups, strengthen integrated chronic care, and reduce geographic inequities. The pandemic experience highlights the importance of resilient systems capable of maintaining continuity during crises. Moreover, the observed role of psychiatric referrals in enhancing follow-up suggests that systematic integration of mental health into geriatric care could improve continuity and long-term outcomes. Telemedicine will remain an important tool, but efforts are needed to overcome digital barriers and address patient perceptions in order to ensure equitable and sustainable adoption.

## 5. Conclusions

This large retrospective study demonstrates that older adults in Poland generate substantial demand for healthcare, with more than ten visits per patient annually on average. Patterns of utilization were shaped by age, chronic disease status, and the systemic disruptions of the COVID-19 pandemic. While patients with chronic diseases received more prescriptions and diagnostic tests, they were less likely to return within 30 days, suggesting a shift toward structured long-term care pathways. Younger seniors tended to return sooner, whereas older adults more often engaged in medium and long-term follow-up.

The pandemic led to a pronounced reduction in visit realization during early waves, followed by a significant recovery in the post-COVID period. Psychiatric referrals were associated with improved short and mid-term continuity of care, underlining the importance of integrating mental health into geriatric services. Telemedicine adoption mitigated some of the access barriers but was limited by digital divides and usability concerns.

Although most associations identified were statistically significant, effect sizes were small, underscoring the need to interpret large datasets with caution and focus on clinically meaningful differences. These findings support the development of resilient, patient-centered models of geriatric care that address multimorbidity, promote equitable access across geographic areas, and incorporate both physical and mental health dimensions.

## Figures and Tables

**Table 1:** Group characteristics based on 738,300 consultations from 42,844 individual patients.

	M	Md	SD	Min–Max
Age	77.53	76.00	9.39	60–111
Number of visits in the last 12 months	10.28	6.00	18.11	0–460
		N	%	
Sex	Female Male	470,547 267,753	63.73 36.27	
Place of visit	Village City	72,197 666,103	90.22 9.78	
Chronic diagnosis	No Yes	716,877 21,423	97.10 2.90	
Visit with referral	No Yes	490,788 247,512	66.48 33.52	
Visit with prescription	No Yes	318,043 420,257	43.08 56.92	
Visit with test order	No Yes	580,176 158,124	78.58 21.41	
Referral to a psychiatrist or psychologist	No Yes	737,621 679	99.91 0.09	
Follow-up after 30 days	No Yes	450,763 287,537	61.05 38.95	
Follow-up after 60 days	No Yes	334,938 403,362	45.37 54.63	
Follow-up after 90 days	No Yes	259,872 478,428	35.20 64.80	
Visit period	Pre-COVID Pre-Pandemic Threat State First Wave Second Wave Third Wave Fourth Wave Post-Pandemic Threat State Post-COVID	32,443 1889 70,908 59,805 74,783 107,323 54,423 336,726	4.39 0.26 9.60 8.10 10.13 14.54 7.37 45.61	

Note. Data reflect 738,300 consultations provided to 42,844 unique patients. Percentages refer to visit-level distributions. M = mean; Md = median; SD = standard deviation.

**Table 2:** Summary of chi-square test results for various analyses.

Analysis Description	χ^2^	df	*p*-Value	Phi Coefficient (φ)	Description
Chronic Disease and Diagnostic Tests	1570.42	1	<0.0001	0.00213	Higher percentage of diagnostic tests ordered for patients with chronic diseases (32.4%) compared to those without (21.1%).
Visit Type and Referral Issuance	1620.51	1	<0.0001	0.00219	More referrals issued during first visits (41.6%) compared to follow-up visits (32.9%).
Visit Completion Status and Prescription Issuance	1048.76	1	<0.0001	0.00194	Prescriptions were issued more frequently during completed visits (63.2%) compared to declared-1 only visits (16.4%).
Chronic Disease and 30-Day Return Visits	2243.76	1	<0.0001	0.00304	Patients without chronic conditions returned within 30 days more frequently (39.4%) than those with chronic conditions (23.4%).
Patient Sex and Healthcare Issuance	12.74	1	0.0004	<0.0002	Women received slightly more interventions than men (57.1% vs. 56.7%).
Place of Medical Appointment	769.96	1	<0.0001	<0.0014	Patients from suburban areas received more interventions (61.8%) than those from urban areas (56.4%).
Patient Sex and Chronic Disease Diagnosis	3.13	1	0.0768	-	No significant relationship between sex and chronic disease diagnosis.

Note. All results are based on chi-square tests of independence. χ^2^ = the chi-square statistic; df = the degrees of freedom; φ = the phi coefficient (effect size). Percentages refer to visit-level distributions. Statistical significance was set at *p* < 0.05. Analyses were conducted on 738,300 consultations.

## Data Availability

Available on justified request.

## Abbreviations

IADL	Instrumental Activities of Daily Living
OR	Odds Ratio
SD	Standard Deviation
χ^2^	Chi-square test statistic
ρ	Spearman correlation coefficient
